# Bilateral Atypical Optic Neuritis With Posterior Scleritis Secondary to Tuberculosis: Challenges in Management

**DOI:** 10.7759/cureus.78387

**Published:** 2025-02-02

**Authors:** Nurul Syahida Abd Wahab, Natashini Rajaratnam, Wan-Hazabbah Wan Hitam, Qi Zhe Ngoo, Shahidatul-Adha Mohamad

**Affiliations:** 1 Ophthalmology and Visual Sciences, Universiti Sains Malaysia, School of Medical Sciences, George Town, MYS

**Keywords:** atypical optic neuritis, optic neuritis, optic neuropathy, posterior scleritis, tuberculosis

## Abstract

Atypical optic neuritis and posterior scleritis can indicate extrapulmonary tuberculosis (TB) caused by *Mycobacterium tuberculosis*. We present a rare case of bilateral atypical optic neuritis accompanied by posterior scleritis secondary to TB. A 36-year-old gentleman presented with a four-day history of blurred vision in both eyes, along with pain during eye movements. His medical history included intermittent low-grade fever and a persistent cough lasting four months, accompanied by significant weight loss of 13 kg over two months. On examination, visual acuity in the right eye was 6/36, while perception of light (PL) was present in the left eye. A positive relative afferent pupillary defect was noted in the left eye, with reduced light brightness and red desaturation observed. Both anterior segments appeared normal. Fundoscopic examination revealed bilateral hyperemic swollen optic discs. Chest radiography showed opacities in the right upper and middle lung fields. MRI of the orbit and brain demonstrated enhancement of the optic nerves in both eyes, along with pronounced posterior scleritis. Sputum analysis for acid-fast bacilli suggested pulmonary involvement. Lumbar puncture results were unremarkable, with normal opening pressure and cytology. The patient was diagnosed with bilateral atypical optic neuritis and posterior scleritis secondary to pulmonary TB. He was promptly started on an antitubercular regimen. Systemic high-dose methylprednisolone was introduced five days later. However, his condition deteriorated to PL in both eyes with pale discs after two weeks. Despite a modified corticosteroid regimen, vision remained poor upon discharge. Atypical optic neuritis may overlap with posterior scleritis and cause severe, sight-threatening conditions. Early diagnosis and comprehensive management of TB are critical for improving outcomes and preventing long-term damage.

## Introduction

Tuberculosis (TB) is a preventable and usually curable disease. It remains a major public health concern in Southeast Asia, with countries like Malaysia facing high incidence rates. According to the World Health Organization, globally, in 2023, an estimated 10.8 million people fell ill with TB (incident cases), a further increase from 10.7 million in 2022 [1]. In Malaysia, TB incidence and mortality rates are estimated at 92 cases and four deaths per 100,000 people, respectively [2]. Ocular involvement occurs in about 1-2% of TB cases. Optic neuropathy in TB can arise from direct infection by mycobacteria or a hypersensitivity reaction to the pathogen. This involvement may present as optic nerve tubercle, papilledema, papillitis, retrobulbar neuritis, neuroretinitis, or optic chiasmatic arachnoiditis [3]. We present a rare case of bilateral atypical optic neuritis with posterior scleritis secondary to TB.

## Case presentation

**Figure 1:**
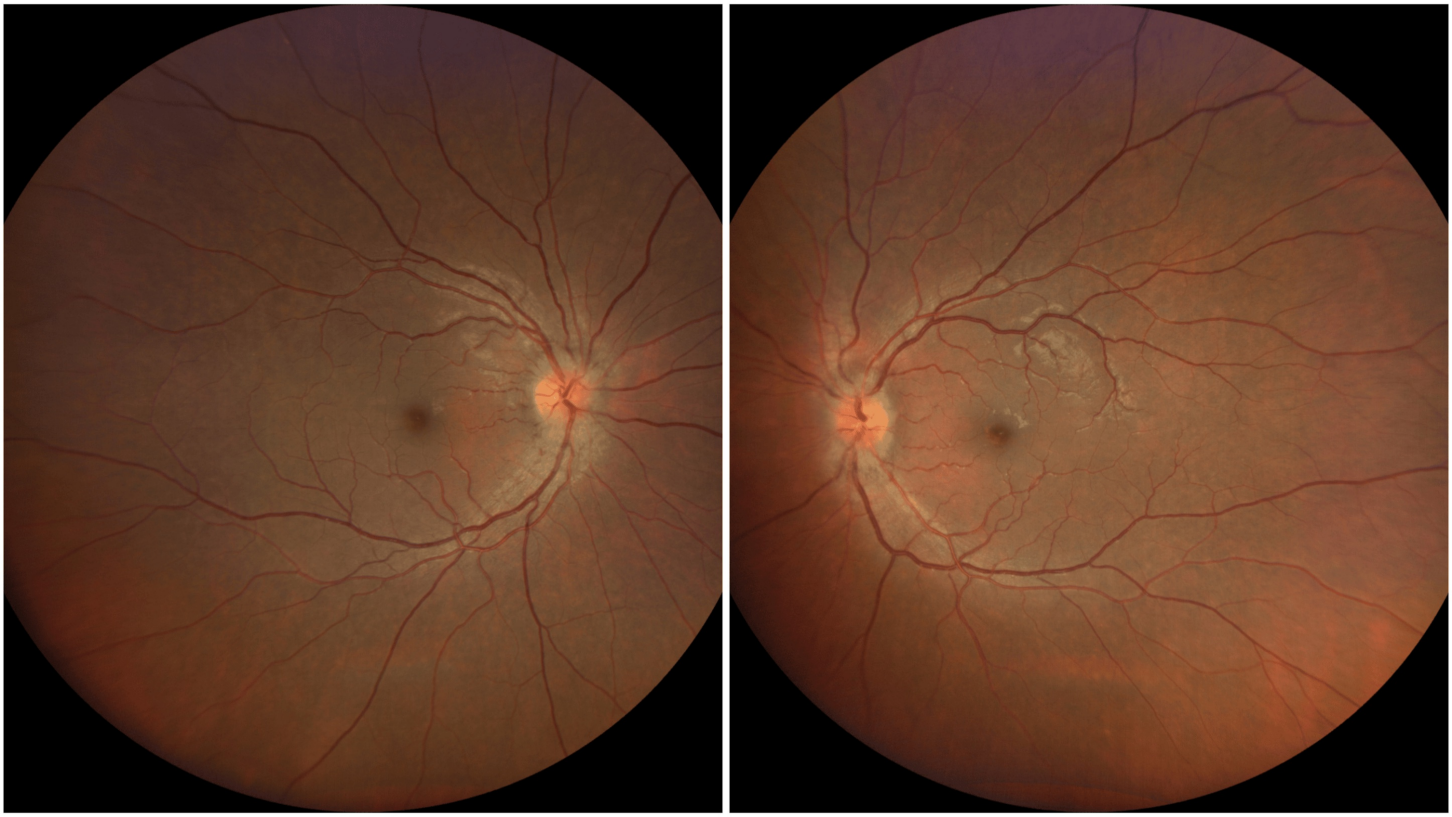
Fundus examination showing both eye hyperemic swollen optic disc

A 36-year-old gentleman with no known medical illness presented with bilateral worsening blurry vision for four days duration associated with right eye pain on movement. He had intermittent low-grade fever and a persistent cough lasting four months. He also experienced a significant weight loss of 13 kg over two months. Otherwise, he had no family history or contact with a TB patient, and he denied high-risk behavior. General examination revealed a healthy, conscious, and well-oriented gentleman; the patient was well-orientated to time, place, and person. Central nervous system (CNS) examinations, including other cranial nerves, were normal. Respiratory examination was also normal. No lymphadenopathy or hepatosplenomegaly was elicited. The cardiovascular system was normal. Visual acuity was 6/36 in the right eye, and perception of light (PL) in the left eye. Relative afferent pupillary defect (RAPD) was positive over the left eye with reduced light brightness and red desaturation. The confrontation test showed that the right eye had a central scotoma, and the left eye could not perform due to poor vision. Both anterior segments were normal. Fundus examination showed a bilateral hyperemic swollen disc (Figure 1). The macula and retina were normal; there was no vitritis, retinitis, or vasculitis. Intraocular pressure was within normal range for both eyes.

Ultrasonography showed a thickened posterior sclera with no choroidal thickening. TB workup was positive for the Mantoux test and sputum acid-fast bacilli (AFB) smear. Blood investigations revealed an elevated erythrocyte sedimentation rate of 90 mm/h and abnormal liver function, with an alanine transaminase level of 114 and an alkaline phosphatase level of 460 (Table 1).

**Table 1:** References value ESR: erythrocyte sedimentation rate, ALT: alanine transaminase, ALP: alkaline phosphatase

	Result	Reference ranges	Unit
ESR	90	<15	mm/hr
ALT	114	10-35	U/L
ALP	460	35-104	U/L

**Figure 2:**
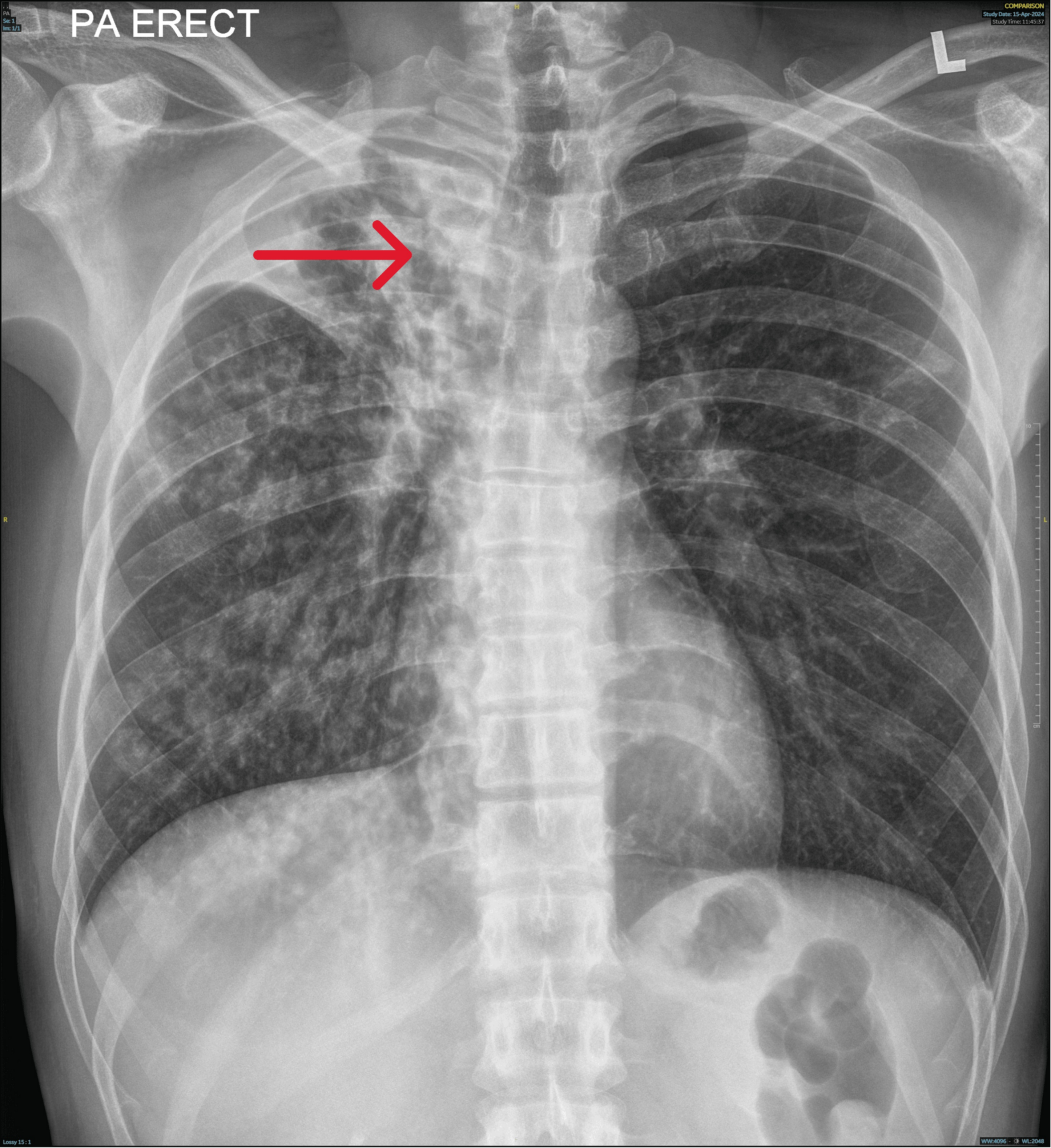
Chest radiograph showing right upper lobe and middle lobe lung opacities (red arrow)

**Figure 3:**
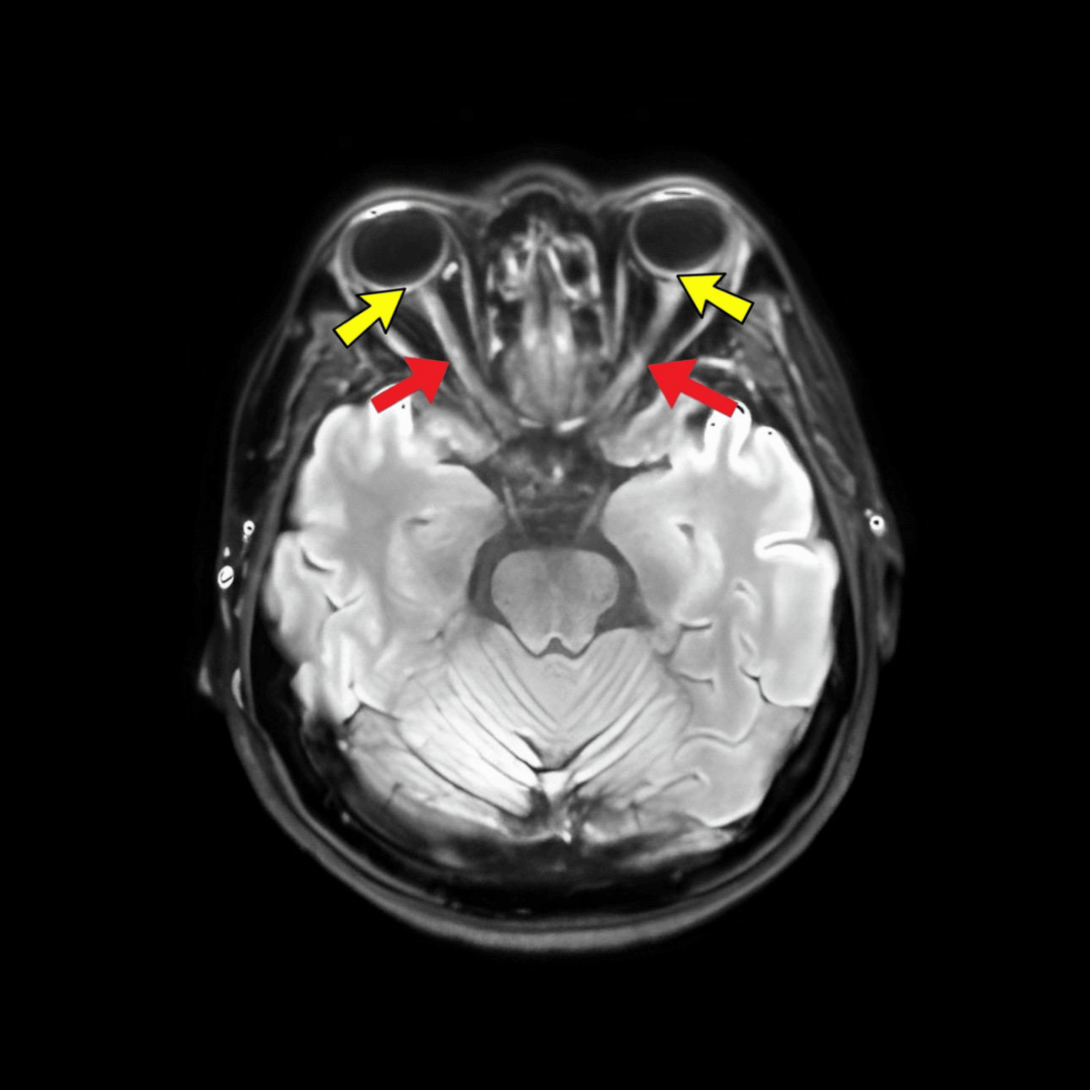
MRI brain and orbit showing enhancement of both optic nerve (red arrows) with posterior scleritis (yellow arrows)

Chest radiography showed opacities in the right upper and middle lung fields (Figure 2). MRI of the orbit and brain demonstrated enhancement of the optic nerves in both eyes with posterior scleritis (Figure 3). Sputum analysis for AFB suggested pulmonary involvement. Lumbar puncture results were unremarkable, with normal opening pressure and cytology.

A diagnosis of bilateral atypical optic neuritis with posterior scleritis secondary to pulmonary TB was made. He was co-managed with the infectious disease team and was started on anti-TB medications. He was started on a directly observed treatment anti-TB regime that included isoniazid, rifampicin, and pyrazinamide with a concurrent prescription of pyridoxine. Five days after initiating anti-TB medications, high-dose intravenous methylprednisolone 1 gram per day was given for five days, followed by an oral prednisolone (1 mg/kg/day) tapering dose. His oral prednisolone was maintained at a dose of 35 mg once daily (OD) for six weeks, then continued with a tapering dose.

**Figure 4:**
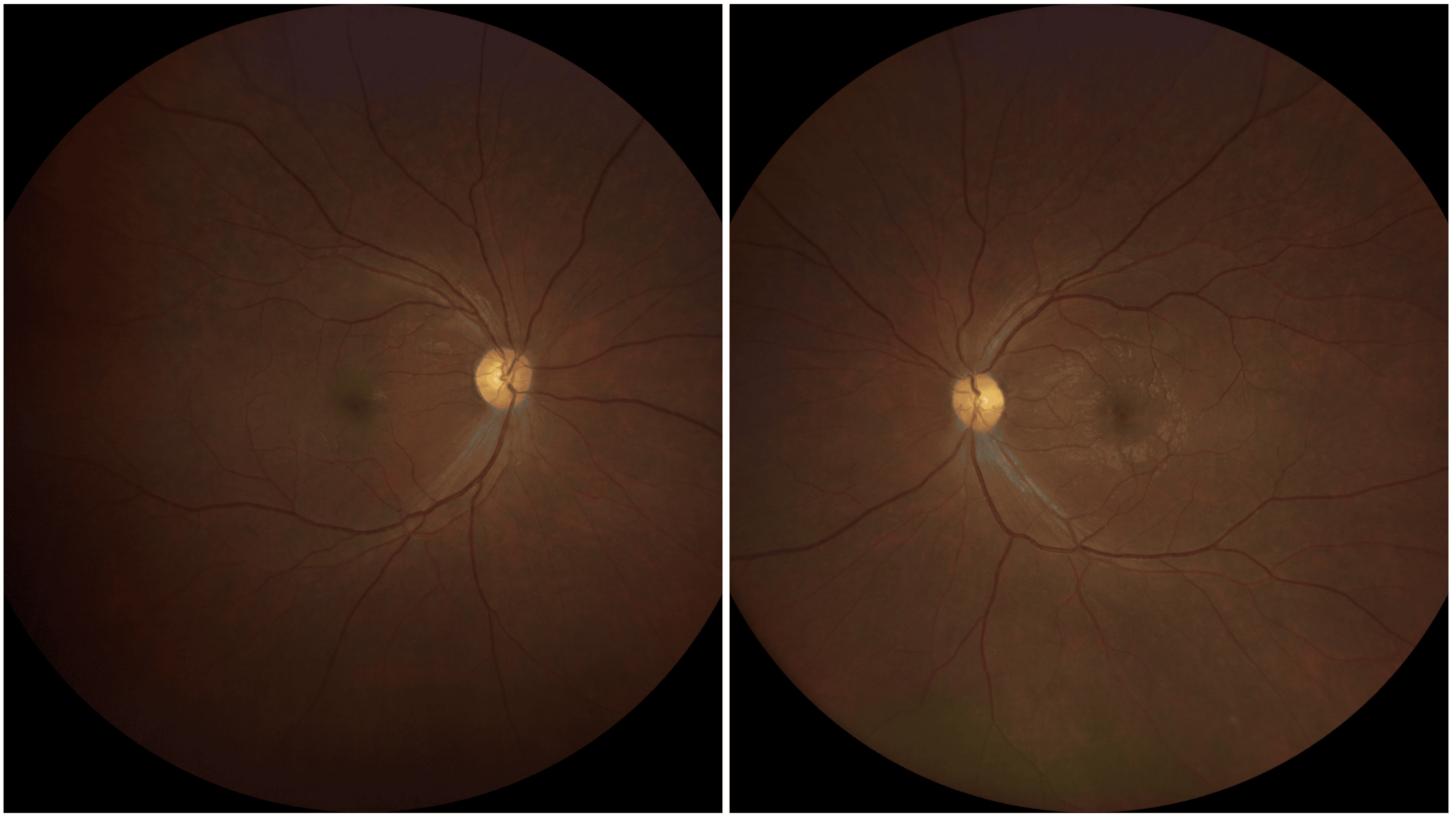
Fundus examination showing both eye optic disc atrophy

During review at two weeks of treatment, it showed that both eyes and vision deteriorated to PL. Fundus examination showed a pale-looking optic disc (Figure 4). Subsequently, his oral prednisolone dose was reduced to 30 mg OD for six weeks and then followed by 25 mg OD for six weeks. Following optic nerve injury, he was also started on vitamin B12 as a neuroprotective agent. Upon the latest follow-up after four months, there was some improvement in vision in the right eye to 6/120 and counting finger in the left eye. Both optic discs remain pale.

## Discussion

Optic neuritis secondary to TB is low compared to more common causes. TB itself is a significant global health issue, particularly in developing countries with high rates of poverty, HIV/AIDS, and limited healthcare infrastructure. Prevalence data for optic neuritis in the context of CNS TB are limited. It is recognized as a very rare manifestation.

The pathophysiology of optic neuritis secondary to TB involves both direct and indirect mechanisms. Directly, TB can cause inflammation of the meninges, which may extend to the optic nerve sheath, leading to compression and impairment of optic nerve function. Additionally, granulomatous lesions known as tuberculomas can form in the brain and affect the optic nerve by direct invasion or inducing localized inflammation [4]. This granulomatous reaction can disrupt the normal function of the optic nerve through inflammation and demyelination. Indirectly, systemic TB can cause a generalized inflammatory response that might contribute to secondary effects on the optic nerve, although this is less direct than primary infection. Furthermore, severe TB can lead to compromised vascular conditions, potentially resulting in ischemic damage to the optic nerve. Increased intracranial pressure from TB meningitis can also affect the optic nerve, contributing to the development of optic neuritis. Thus, while optic neuritis due to TB is rare, it typically arises from a complex interplay of direct infection or inflammation and indirect systemic effects.

Optic neuritis secondary to TB is a rare but serious complication that can present with a range of clinical manifestations. The primary symptom is often a significant loss of vision, which may be accompanied by visual field defects such as central scotomas or peripheral vision loss. Patients may also experience eye pain, particularly with movement, although this symptom is not always present. Color vision impairment, where patients have difficulty distinguishing colors, can also occur [5]. Disc edema may be observed during a fundoscopic examination, but in some cases, the optic disc may appear normal, especially in the condition's early stages. On presentation, our patient had bilateral disc edema with a central scotoma.

Systemic symptoms of TB, such as fever, night sweats, weight loss, and cough, can offer important clues to the presence of the infection. For instance, our patient exhibited fever, cough, and significant weight loss, which led to the confirmation of TB through a positive sputum AFB test.

Diagnostic evaluation for TB affecting the central nervous system often includes an MRI of the brain to detect tuberculomas or other signs of CNS TB. In our patient’s case, the MRI of the orbit and brain revealed enhancement of the optic nerves in both eyes and posterior scleritis, but no brain lesions were found. Additionally, a lumbar puncture was performed to analyze cerebrospinal fluid for abnormalities indicative of tuberculous meningitis or other CNS manifestations; the lumbar puncture results were normal.

Posterior scleritis is a relatively uncommon cause of scleral inflammation, and tubercular posterior scleritis is even rarer. Prior to this report, only four cases of tuberculous posterior scleritis had been documented in the literature, mostly of the nodular anterior scleritis type [6]. TB-associated posterior scleritis may present without anterior segment symptoms and typically responds well to a treatment regimen combining antitubercular therapy with corticosteroids [7]. Posterior scleritis is associated with a higher risk of permanent visual loss, ranging between 30% and 50%, as well as complications and disease recurrence [8].

In our case, the patient exhibited optic disc edema with subretinal fluid, which is a sight-threatening disorder and warrants aggressive systemic therapy. Posterior scleritis exhibits a range of manifestations, and various fundus signs have been observed in individuals with tuberculous posterior scleritis [9].

Management of TB involves treating the underlying infection with a regimen of antitubercular medications, primarily developed in the mid-20th century. The fourth edition of the clinical practice guidelines for TB management recommends the standard 2EHRZ/4HR regimen as the most effective treatment, providing the highest success and lowest relapse rates. Alternative regimens are reserved for cases where the standard treatment is contraindicated or poorly tolerated.

Corticosteroids may be used to reduce inflammation and improve visual outcomes but are typically administered alongside TB treatment rather than as a standalone therapy. In this case, the patient was initially treated with the SHM regimen for five days, followed by SHMR for two days, and then SHRZ for two months. The patient is currently in the maintenance phase with the HR regimen (S = streptomycin, H = ethambutol, M = moxifloxacin, R = rifampicin, Z = pyrazinamide).

Additionally, five days after starting anti-TB medications, the patient received high-dose intravenous methylprednisolone (1 gram per day) for five days, followed by oral prednisolone (1 mg/kg/day) with a tapering dose. Treatment typically combines antitubercular medications to address the TB infection and corticosteroids or other anti-inflammatory agents to manage ocular inflammation.

The prognosis for optic neuritis secondary to TB can vary significantly based on the timing and effectiveness of treatment for both the optic neuritis and the underlying TB infection. Early diagnosis and appropriate management with antitubercular therapy and supportive care can improve many patients' visual function. However, the degree of recovery largely depends on the severity of optic nerve damage and any additional complications.

In some cases, patients may achieve partial or complete vision recovery, though this process can be slow and variable. If TB is not adequately treated, complications such as persistent or worsening vision loss due to ongoing inflammation or damage to the optic nerve can occur. Furthermore, patients may face additional challenges from other TB-related complications, such as tuberculous meningitis or brain tuberculomas, which can further complicate the clinical picture and affect overall outcomes.

Long-term management often requires ongoing monitoring and additional treatments to address residual symptoms or complications. Early diagnosis and a comprehensive treatment approach are essential for improving prognosis and reducing the risk of serious complications.

## Conclusions

This report details a rare case of bilateral optic neuritis occurring concurrently with isolated posterior scleritis due to pulmonary TB. Ocular TB can present with a wide range of symptoms and impact various parts of the eye, leading to diverse clinical manifestations. Optic neuritis may overlap with posterior scleritis, which may cause severe, sight-threatening conditions. Early diagnosis and comprehensive management of TB are critical for improving outcomes and preventing long-term damage. Regular monitoring and follow-up are essential for effectively managing these complications and adjusting treatment. In acute cases, initiating systemic steroids and antimicrobial therapy immediately is advisable to minimize severe damage to the optic nerve.
